# Volumetric Modulated Arc Therapy Versus Dynamic Conformal Arc Therapy for Single Isocenter Stereotactic Radiotherapy of Multiple Brain Metastases

**DOI:** 10.3390/bioengineering13020207

**Published:** 2026-02-12

**Authors:** Theodoros Stroubinis, Maria Giannopoulou, Despoina Stasinou, Michalis Psarras, Anna Zygogianni, Maria Protopapa, Vassilis Kouloulias, Kalliopi Platoni

**Affiliations:** 1 Department of Radiation Oncology and Stereotactic Radiosurgery, Mediterraneo Hospital, 16675 Athens, Greece; despoinast95@gmail.com (D.S.); mixpsarras@gmail.com (M.P.); merryprotopapa@yahoo.fr (M.P.); 2 Department of Applied Medical Physics, Medical School, National and Kapodistrian University of Athens, Attikon University Hospital, 12462 Athens, Greece; mara.k.giannopoulou@gmail.com; 3 Laboratory of Clinical and Radiation Oncology, Medical School, National and Kapodistrian University of Athens, Attikon University Hospital, 12462 Athens, Greece; annazygo1@yahoo.gr (A.Z.); vkouloul@ece.ntua.gr (V.K.)

**Keywords:** SRS, radiosurgery, brain metastases, single isocenter, VMAT, DCA, optimized DCA, plan complexity, dosimetric parameters, quality metrics

## Abstract

Introduction: Stereotactic radiosurgery is a highly precise radiotherapy technique widely used for the management of brain metastases. While VMAT enables highly conformal dose distributions, it is often associated with increased plan complexity and longer delivery times. Optimized dynamic conformal arc therapy (OptDCA) represents a less complex alternative that may achieve comparable dosimetric performance. In this retrospective study, dosimetric quality, deliverability, and plan complexity of VMAT and OptDCA were compared for single-isocenter SRS of multiple brain metastases. Materials and Methods: Thirty patients previously treated with VMAT were randomly selected and replanned using OptDCA with identical beam arrangements. Plan quality was evaluated using the Paddick conformity index, gradient index, target coverage, MUs, and brain V12Gy and V20Gy. Deliverability was assessed using gamma passing rates, and plan complexity was quantified using multiple complexity metrics. Results: VMAT achieved a slightly higher CI (0.72 vs. 0.71) but required a higher number of MUs (5376 vs. 4820), while no significant differences were observed in GI or target coverage. OptDCA demonstrated significantly higher GPR (median 96.95% vs. 91.1%) and consistently lower plan complexity. Significant correlations were observed between GPR and several complexity metrics for both techniques. Conclusion: Overall, OptDCA provides comparable plan quality to VMAT, while offering improved deliverability and reduced complexity, making it a viable alternative technique.

## 1. Introduction

Stereotactic radiosurgery (SRS) has become an integral component of modern radiation oncology, offering highly precise, ablative radiation delivery for intracranial targets while minimizing dose to surrounding healthy brain tissue. Advances in treatment planning algorithms, beam modulation techniques, and delivery systems have enabled the safe administration of high radiation doses in one or a limited number of fractions, expanding the clinical applicability of SRS across a wide range of indications [1,2]. A defining characteristic of SRS is the requirement for steep dose gradients at the target margins, which places substantial demands on treatment planning strategies and delivery accuracy [3].

One of the most common clinical applications of SRS is the management of brain metastases (BMs), which represent the most frequent malignant tumours of the central nervous system. Epidemiological studies indicate that between one-fifth and two-fifths of individuals with cancer will develop brain metastases during the course of their disease [4]. At initial diagnosis, approximately 70% of patients present with a solitary lesion, although the number of metastases varies depending on the primary tumour type. Lung cancer is the predominant source (approximately 40–50%), followed by breast cancer (15–25%), melanoma (5–20%), and renal cell carcinoma (5–10%) [5,6].

A variety of radiosurgery techniques exist, each capable of delivering a high ablative dose with a steep dose gradient through distinct technical approaches. In LINAC-based SRS, this is typically achieved using multiple intersecting non-coplanar dynamic conformal arcs (DCA), or volumetric modulated arc therapy (VMAT) with a set of intensity-modulated beams [7].

VMAT is a treatment delivery method that combines simultaneous modulation of the MLC aperture, radiation dose rate, and gantry rotation speed to produce highly conformal dose distributions. In radiosurgery settings, treatment plans often employ multiple VMAT arcs with three or more couch angles to maximize target dose conformity and ensure a steep dose gradient at the target boundary [8].

Plan complexity determines the amount of modulation and technical effort required to achieve accurate dose delivery and is typically characterized based on machine parameters and plan attributes such as fluence, MLC aperture, position and displacement, gantry speed and dose rate variations, and monitor units (MUs) [9]. While greater complexity will enhance conformity, it can reduce efficiency and enhance quality assurance (QA) discrepancy risk [10,11]. Several metrics, such as the modulation complexity score (MCS) and aperture shape-based indices, have been put forward to quantify this balance [9,12]. Existing evidence is in favour of the fact that SRS/VMAT plans are highly complex, with studies attributing undue modulation to reduced deliverability and decreased gamma passing rates [13,14,15].

The complexity of delivering VMAT treatment plans can vary considerably; therefore, it is crucial to quantify this complexity and investigate the potential relationship between treatment deliverability and the outcomes of pre-treatment, patient-specific quality assurance assessments. Reducing delivery complexity in VMAT radiosurgery may be beneficial, provided that comparable plan quality is maintained [2].

A technique that can reduce plan complexity is the DCA. In this technique, we can deliver very high doses with precision by dynamically shaping the MLC aperture to nearly exactly match the target volume during gantry rotation, while maintaining a constant dose rate and gantry speed. It employs a series of conformal arcs that continuously adjust the MLC leaves to the tumour’s shape so that efficient dose delivery with lesser plan complexity may be achieved as compared with highly modulated approaches such as VMAT [16,17]. DCA usually attains good conformity to spherical or regularly shaped lesions, whereby it is challenged with oddly shaped targets or multiple metastases, for which advanced modulation techniques such as VMAT can offer better dose conformity [18].

Considering these factors, one approach is to combine the DCA and VMAT techniques by importing the DCA plan into the TPS photon optimizer (PO) module. In these optimized DCA (OptDCA) plans, certain parameters can be used to reduce plan complexity and modulation, thereby increasing delivery efficiency while maintaining dosimetric accuracy [19,20]. One such parameter is the aperture shape controller, which limits the allowed leaf movement to create smoother MLC trajectories that reduce unnecessary modulation, potentially decreasing treatment time [21]. Similarly, MU objectives help keep the limits reasonable, avoiding an excessive number of MUs that could increase complexity and affect deliverability [2].

The aim of this study is to compare OptDCA with VMAT techniques for single-isocenter stereotactic radiosurgery of multiple brain metastases, not only in terms of plan quality, dosimetric accuracy, and deliverability but also treatment efficiency and robustness with the aim of setting up scenarios where OptDCA can be used as a simpler yet effective alternative to VMAT.

## 2. Materials and Methods

**Scheme 1:**
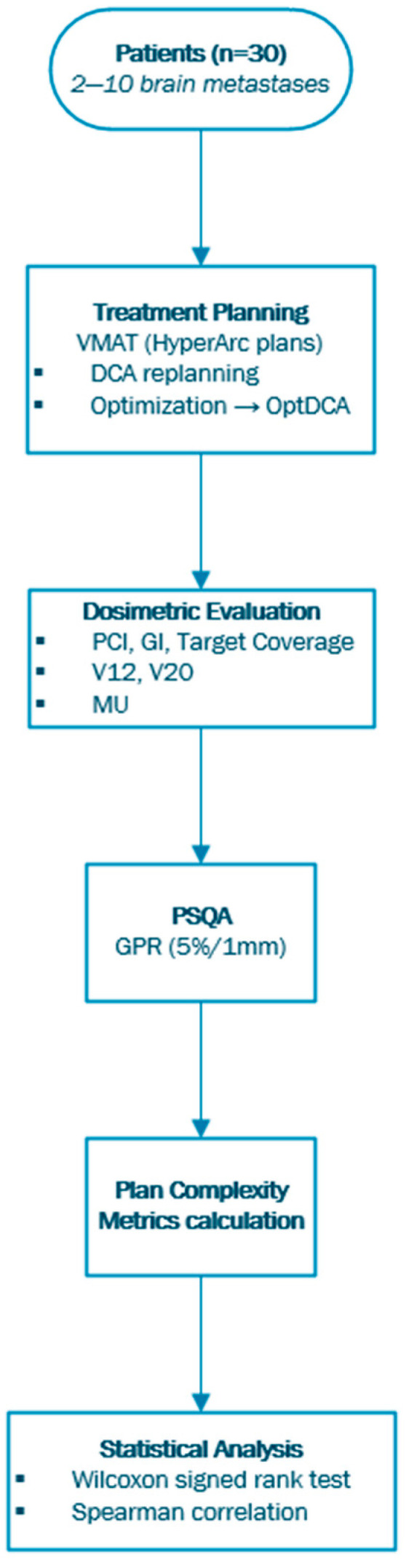
Flowchart of the methodology structure.

The methodology of this study is depicted in the flowchart of Scheme 1.

### 2.1. Patients

Thirty patients were retrospectively and randomly selected. Each patient presented with 2 to 10 brain metastases, as shown in Table 1 and Table A1, with individual lesion volumes ranging from 0.02 to 14.6 cc. These patients in our clinic had initially been treated with VMAT-based therapy using a single-isocenter SRS technique.

### 2.2. HyperArc Treatment Planning

Treatment plans were generated with the HyperArc algorithm of the eclipse treatment planning system (TPS) (Varian, version 15.6) and was delivered on the Edge linear accelerator with a high-definition 120-leaf multileaf collimator (HD 120 MLC; Varian Medical Systems, Palo Alto, CA, USA). For every patient, 6 MV flattening filter-free (FFF) photon beams were used, and the maximum dose rate was 1400 MU/min. The planning geometry was automatically established by the HyperArc algorithm, placing the isocenter at the barycenter of the metastatic lesions, choosing the optimal collimator angle for each arc [22], and setting four arcs per plan—one coplanar and three noncoplanar, including couch rotations of 315°, 45°, and either 90° or 270° based on patient anatomy [3].

### 2.3. DCA Treatment Planning

All the above HyperArc plans were replanned using the DCA technique, with their geometry and energy being kept the same as the initial plan. First, in the DCA plans, the MLCs were adjusted so that they maintain a 0.1 cm distance from all targets during the arc rotation, with a field weight of 1. For each patient, dose normalization was performed to ensure that all plans had the same target volume coverage with VMAT plans.

Using the PO algorithm, the MLC aperture shape controller was applied, assigning high priority to maintaining the shape of the aperture close to the PTV. In combination, the monitor unit (MU) objective was used to keep the number of monitor units as low as possible.

### 2.4. Data Analysis

For the comparison of the techniques, plan quality indices of the Paddick conformity index (PCI) [23] and gradient index (GI) were initially calculated, using the following formulas:

(1) $$PCI=\frac{{{TV}_{PIV}}^{2}}{TV\cdot PIV}$$

- TV_PIV_: Target volume covered by the isodose curve of the prescribed dose.
- PIV: Volume covered by the isodose corresponding to the prescribed dose.
- TV: Total volume of the target.

(2) $$GI=\frac{V_{50\%}}{V_{100\%}}$$

- V**_50%_**: The volume of tissue receiving 50% of the prescribed dose.
- V**_100%_**: The volume of tissue receiving 100% of the prescribed dose.

Furthermore, from the DVH, the irradiation of healthy brain tissue was measured using the V_12_ and V_20_ indices, with V_12_ representing the absolute volume of brain tissue receiving over 12 Gy and V_20_ representing the absolute volume receiving over 20 Gy, respectively, along with the overall MU value for each treatment plan.

Treating multiple targets with linac-based SRS introduces uncertainties that can impact the plan’s deliverability. Therefore, patient-specific quality assurance (PSQA) is crucial for verifying whether the plan meets our delivery criteria. The PSQA process was performed using the Octavius 4D Modular Phantom (O4D) (PTW, Freiburg, Germany), which is a cylindrical phantom made of water-equivalent plastic (density: 1.05 g/cm^3^) [24]. It features a central insert designed to accommodate a detector array. In this study, the 1600 SRS detector array was used in combination with the Octavius 4D Phantom. Dose measurement and analysis were performed using the Verisoft software v8.1 (PTW, Freiburg, Germany). Verisoft aggregates data from multiple measurements and enables 3D dose reconstruction for both coplanar and non-coplanar beam geometries. The calculated gamma passing rates (GPRs) were recorded for the 5%/1 mm criteria with a threshold of 10%.

To evaluate the complexity of plans for both techniques and compare them, we used UComX v1.1, a previously verified MATLAB v23.2-based software package [25] to calculate complexity metrics. These metrics are classified into two categories [26]:

Deliverability metrics assess the machine’s capability to accurately deliver the treatment as planned, considering variations in mechanical parameters (gantry, MLC) and dosimetric parameters (dose rate, MU). These metrics may be evaluated individually or in combination, and their applicability depends on the technique employed. Τhe metrics considered in our study were: plan irregularity (PI), average leaf pair opening (ALPO), leaf speed (LS), aperture area variability (AAV), leaf sequence variability (LSV), modulation complexity score (MCS) [26].

Accuracy metrics are designed to quantify the parameters most likely to affect the precision of dose calculation, arising from limitations in machine modelling and algorithmic inaccuracies within the TPS. Τhe metrics considered in our study were: small aperture score (SAS), edge metrics (EM), equivalent field size (EFS) [26]. The aforementioned metrics are shown in Table A2.

A Wilcoxon signed-rank test was conducted (IBM SPSS, version 29) to evaluate differences in dosimetric parameters and complexity metrics between HyperArc and OptDCA. A *p*-value below 0.05 was regarded as statistically significant. Additionally, we examined the potential relationship between complexity metrics and GPR for both techniques using the Spearman test.

## 3. Results

### 3.1. Plan Quality and Dosimetric Comparison

Dosimetric and delivery-related metrics were used to compare plan quality, normal brain sparing, and treatment efficiency between HyperArc and OptDCA. The two techniques achieved comparable target coverage and dose gradient, with small but statistically significant differences in conformity and normal brain dose.

According to Table 2, HyperArc plans and OptDCA plans showed similar GI and target coverage, with no statistically significant differences. Regarding PCI, HyperArc demonstrated values comparable to OptDCA, with a statistically significant difference (*p* = 0.011). HyperArc plans, compared to OptDCA, had lower values of V_12_ and V_20_, with statistically significant differences in both cases (*p* < 0.001 and *p* = 0.001, respectively). Finally, concerning MUs, HyperArc plans resulted in higher values, and a statistically significant difference was found (*p* = 0.007).

### 3.2. PSQA Results

OptDCA plans demonstrated statistically significant superior deliverability compared with HyperArc, as reflected by higher GPRs.

**Figure 1:**
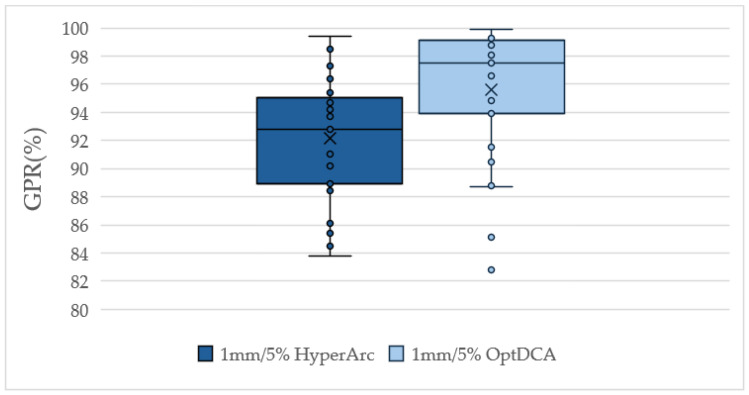
Boxplots of GPR with criteria 1 mm distance-to-agreement and 5% dose difference. The dark blue refers to HyperArc plans, and the light blue refers to OptDCA plans.

Figure 1 presents a boxplot of GPR to assess the treatment’s deliverability. The HyperArc plans demonstrated a lower median GPR of 91.1% (range: 83.8–99.4), whereas the corresponding OptDCA plans had a median of 96.95% (range: 82.8–99.9).

### 3.3. Plan Complexity Metrics

The complexity metrics were computed for the plans of both techniques, and subsequently, an analysis was conducted to determine whether statistically significant differences existed between these techniques. The results indicated that all metrics exhibited statistically significant differences between the two techniques (*p* < 0.001), consistently indicating higher modulation demands for HyperArc compared with OptDCA.

**Figure 2:**
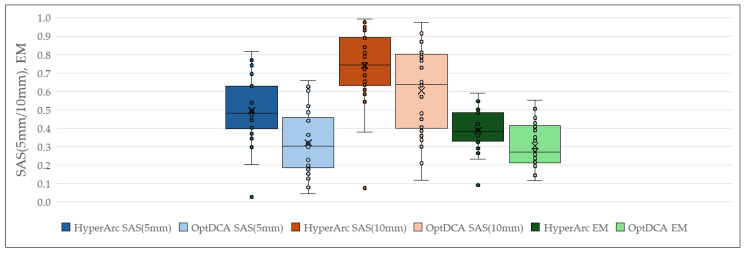
Boxplots of accuracy metrics (SAS, EM). In blue for SAS (5 mm) metrics, in orange for SAS (10 mm) metrics, and in green for EM metrics. The dark colours refer to HyperArc plans, and the light colours refer to OptDCA plans.

Regarding the accuracy metrics, as illustrated in the boxplots of Figure 2, both in SAS (with criteria of 5 mm and 10 mm) and in EM, the HyperArc technique (darker colours) demonstrated higher values than OptDCA (lighter colours), indicating increased complexity. More specifically, it exhibited a median value of 0.48 (range: 0.03–0.81) for SAS (5 mm) and 0.74 (range: 0.08–0.99) for SAS (10 mm) for the HyperArc technique versus 0.31 (range: 0.04–0.65) and 0.63 (range: 0.12–0.66) for the OptDCA one. Likewise, Hyperarc plans showed a median value for EM of 0.38 (range: 0.09–0.59) compared to 0.27 (range: 0.12–0.55) for OptDCA, indicating that HyperArc exhibits more pronounced geometric complexity at the edge, as well as a higher frequency of small MLC openings.

**Figure 3:**
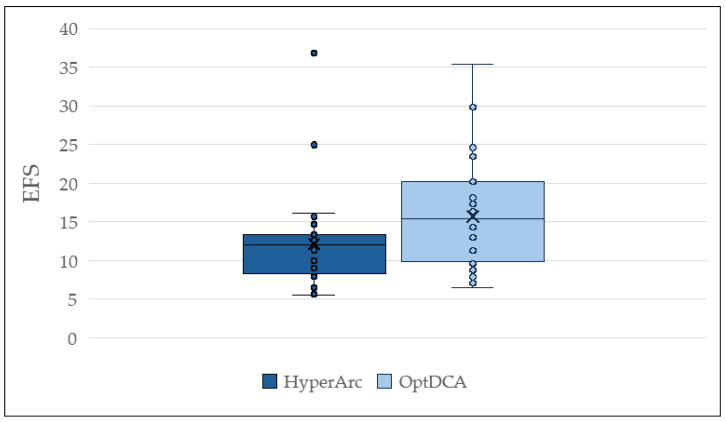
Boxplots of accuracy metrics (EFS). In dark blue for HyperArc plans and light blue for OptDCA plans.

Similarly, in the subsequent metric examined, EFS, the HyperArc technique exhibited lower values, associated with increased complexity, with a median of 11.97 (range: 5.56–36.79) compared to 15.35 (range: 6.43–35.4), as shown in Figure 3.

**Figure 4:**
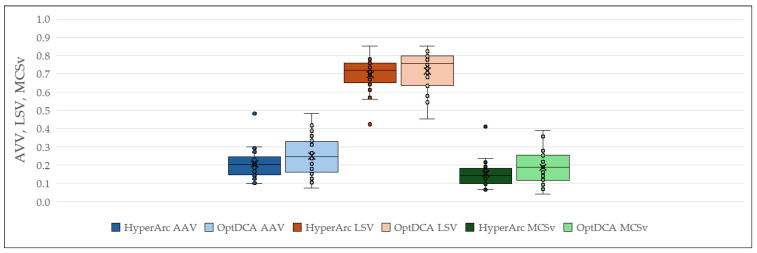
Boxplots of deliverability metrics (AVV, LSV, MCS). In blue for AVV metrics, orange for LSV metrics, and green for MCS metrics. Dark colours represent HyperArc plans, while light colours represent OptDCA plans.

Regarding the deliverability metrics, the HyperArc technique showed lower values for AAV, LSV, and MCS, indicating increased complexity. Specifically, the median AAV was 0.202 (range: 0.098–0.482) for HyperArc compared to 0.244 (range: 0.074–0.484), suggesting slightly higher variation of the MLC at each control point with HyperArc. For LSV, HyperArc values were 0.719 (range: 0.424–0.853) versus 0.754 (range: 0.453–0.851) for OptDCA. For MCS, HyperArc scored 0.144 (range: 0.066–0.411) compared to 0.189 (range: 0.041–0.390), as also visually depicted in Figure 4.

**Figure 5:**
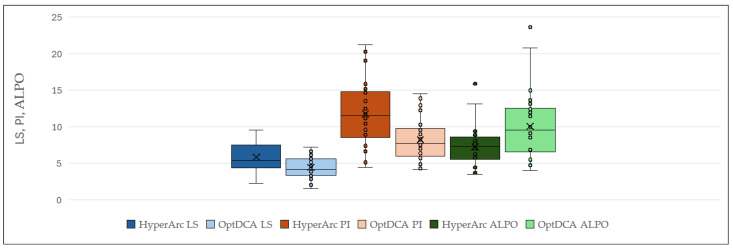
Boxplots of LS, PI, and ALPO. In blue for LS metrics, orange for PI metrics, and green for ALPO metrics. Dark colours represent HyperArc plans, while light colours represent OptDCA plans.

Finally, as Figure 5 shows, the HyperArc technique exhibited higher median values for the LS and PI metrics and a lower median value for ALPO, once again highlighting the technique’s pronounced complexity. More particularly, the values were 5.4 (range: 2.28–9.56), 11.49 (range: 4.42–21.2), and 7.31 (range: 3.48–15.92), respectively, with the corresponding OptDCA values 4.16 (range: 1.54–7.18), 7.7 (range: 4.16–14.5), and 9.56 (range: 4.02–23.65). The HyperArc technique requires higher MLC leaf speed and complex geometric apertures, thereby increasing its complexity due to the pronounced mechanical demands.

### 3.4. Correlation Between Plan Complexity and Deliverability

**Figure 6:**
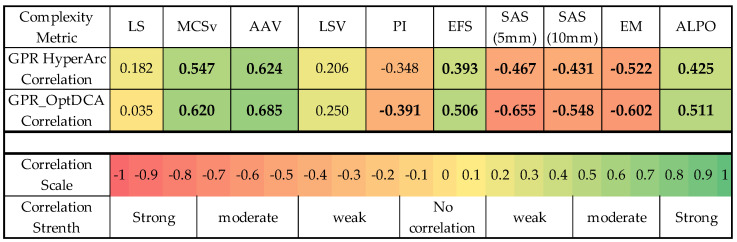
Heatmap for the ρ Spearman test for both techniques. Bold values indicate statistically significant correlations.

Multiple complexity metrics showed statistically significant correlations with gamma passing rates for both techniques. As demonstrated by the heatmap in Figure 6, statistically significant moderate positive correlations were identified for the HyperArc and OptDCA plans regarding the MCSv (ρ = 0.547, ρ = 0.620 with *p* = 0.002, *p* < 0.001, respectively) and AAV (ρ = 0.624, ρ = 0.685 with *p* < 0.001, *p* < 0.001, respectively) metrics. Furthermore, statistically significant weak positive correlations were observed for EFS (ρ = 0.393 with *p* = 0.032) and ALPO (ρ = 0.425 with *p* = 0.019) in the HyperArc plans. Additionally, a statistically significant moderate negative correlation was noted for EM (ρ = −0.522 with *p* = 0.003), accompanied by statistically significant weak negative correlations for SAS at 5 mm and 10 mm (ρ = −0.467, ρ = −0.431 with *p* = 0.009, *p* = 0.017, respectively). For the OptDCA plans, statistically significant moderate positive correlations were also observed for EFS (ρ = 0.506 with *p* = 0.004) and ALPO (ρ = 0.511 with *p* = 0.004), alongside statistically significant moderate negative correlations for EM (ρ = −0.602 with *p* < 0.001) and SAS at 5 mm and 10 mm (ρ = −0.655, ρ = −0.548 with *p* < 0.001, *p* = 0.002, respectively). Lastly, a statistically significant weak negative correlation was identified for the PI metric (ρ = −0.391 with *p* = 0.033).

## 4. Discussion

A particular challenge in the SRS treatment of multiple brain metastases is the ability to irradiate multiple targets at once, which may vary in size and be located in different regions of the brain. VMAT single-isocenter approaches can save a lot of time during treatment, but they are often more complex and challenging in regard to deliverability. Therefore, finding other techniques that allow for simpler treatment plans is essential. For that reason, we generated and compared DCA technique plans that are simpler than VMAT because they do not require optimization. However, the DCA method alone could not set up plans that were clinically acceptable. The DCA plans were recalculated using an optimizer to generate OptDCA plans by applying parameters such as the aperture shape controller and MU objective in order to limit plan modulation. This allows us to generate plans which are clinically acceptable and are essentially comparable with the original HyperArc concerning dosimetry and quality plans.

Especially, the conformity index analysis revealed that the HyperArc technique registered a median PCI of 0.72 (range 0.41–0.90), marginally higher than for OptDCA’s 0.71 (range 0.15–0.91), with the difference reaching statistical significance (*p* = 0.011). This is in agreement with the expected performance of VMAT, whose higher degree of modulation allows a closer fit of the isodose distribution around irregular or larger lesions [27]. However, the difference was relatively modest, radiating the ability of the OptDCA approach, especially with the use of aperture shape controller and MU constraints, to achieve adequate conformity despite its lower modulation. It should also be emphasized that for very small lesions (<1 cc), PCI values are less reliable, as described by Stanley et al. [28], which may partly explain the overlap observed between the two techniques.

Comparison of the GΙ uncovered no statistically significant difference between the techniques, with HyperArc yielding a median GI of 4.09 (range 2.52–89.53) and 3.88 (range 1.40–57.67) for OptDCA (*p* = 0.181). While VMAT through its higher modulation tends to have a slightly steeper dose fall-off, our data reveal that OptDCA can achieve comparable or even superior GI values. This is due to the more gradual and more consistent aperture transitions of the DCA-based solution, which tend to reduce mid-dose spillage, as Jiang et al. also found [29]. This means HyperArc tends to show better conformity for small complex or irregular targets, while OptDCA can show the same gradient performance but with the additional benefit of reduced plan complexity.

The two approaches differed the most in low-to-intermediate dose spillage. HyperArc reduced V_12_ and V_20_ volumes significantly relative to OptDCA: V_12_ was 6.95 cc (range 3.18–17.4) vs. 9.33 cc (range 3.64–25.26; *p* < 0.001), and V_20_ was 2.56 cc (range 0.85–6.01) vs. 2.73 cc (range 0.88–8.1; *p* = 0.001). Although OptDCA values were still below the 10 cc threshold associated with an increased risk of radionecrosis, the greater low-dose spread is consistent with its lesser degree of modulation. These findings show that HyperArc’s strongly modulated apertures provide tighter shaping around clustered lesions, thereby sparing more normal tissue, whereas OptDCA maintains acceptable values with the benefit of reduced complexity. Several studies have evaluated the differences between VMAT and DCAT techniques for SRS cases. DCAT is a similar technique to OptDCA since it is a forward-planned technique that delivers the dose using continuously rotating gantry arcs with MLCs conforming to the target shape. Hofmaier et al. [30] reported median V_12_ values of 2.1 cc for DCAT vs. 3.1 cc for VMAT; however, they stated that most of their targets were small in size with a median volume of 0.8 cc and with high sphericity. Accordingly, Chambrelant et al. reported V_21_ values were lower for the VMAT technique and there was a correlation between the values and the size of metastases, as statistically significant differences between DCAT and VMAT regarding PTV coverage were found only for targets smaller than 10 cc [31].

OptDCA required substantially fewer monitor units: 4735 (range 1408.8–9562.6) compared to 5473 (range 1883.5–9497.7) for HyperArc (*p* = 0.007). This ~13.5% MU reduction saves beam-on time, which may minimize patient movement and maximize comfort. These results are consistent with the literature. Velten et al. also concluded that VMAT plans for certain brain metastases required a median difference between 18% and 24% more MUs than DCA-based plans [32], and Hofmaier et al. presented median MU values of over 5800 in VMAT and about 4500 in DCAΤ [30]. Our findings are therefore to be anticipated, demonstrating that reduced modulation in OptDCA translates into lower demands for MUs and improved treatment efficiency without loss of target coverage.

For gamma analysis, OptDCA was superior to HyperArc. Using the 5%/1 mm criteria, OptDCA achieved higher pass rates, namely, 96.95% (range 82.2–99.9) versus 91.1% (range 83.8–99.4–99.4) for HyperArc (*p* < 0.001). These findings unequivocally show that decreased modulation complexity does lead to enhanced deliverability. Lobb et al. reported no correlation between different techniques and GPR, with a median GPR of 96.81% for VMAT versus 99.28% for DCA using 2%/1 mm criteria, but the study focused on single-target SRS compared to our single-isocenter multi-focal SRS study [2]. Other groups have reported similar findings to ours, with Bokrantz et al. showing that in SBRT lung cases VMAT showed higher complexity in terms of evaluation of MCS and LT complexity metrics [33]. Our results add to the evidence that OptDCA’s simpler and more symmetrical aperture shapes allow for more stable and reproducible delivery in stereotactic treatments

Overall, across measures, HyperArc plans were more complex. For example, HyperArc exhibited higher leaf speed (median 5.4 vs. 4.16 cm/s), higher plan irregularity (11.49 vs. 7.7), and lower ALPO (7.31 vs. 9.56), all statistically significant (*p* < 0.001). Similarly, accuracy-related measures also showed higher small aperture scores (SAS 5 mm: 0.48 vs. 0.31; SAS 10 mm: 0.74 vs. 0.63) and edge metrics (0.38 vs. 0.27) for HyperArc, again indicating higher modulation. These results agree with Sağlam et al. [34], who observed that constraining aperture and MU limits in DCA reduce complexity while providing satisfactory conformity. Most complexity indexes, 6 out of 10 in Hyperarc cases and 7 out of 10 in OptDCA cases, correlated significantly with GPR, which would suggest that the general prediction of modulation is reflective of QA outcomes. The most reliable predictors were AAV (ρ = 0.624, *p* < 0.001) and MCSv (ρ = 0.547, *p* = 0.002) for Hyperarc plans and AAV (ρ = 0.685, *p* < 0.001) and SAS 5 mm (ρ = −0.655, *p* < 0.001) for OptDCA plans. These emphasize the importance of having well-open apertures since smaller openings are prone to dosimetric inaccuracy [12].

Our results suggest that while HyperArc has superior sparing of normal brain tissue, OptDCA offers equivalent conformity and GI, with significantly less MUs, higher GPRs, and lower complexity. These differences arise from the fundamentally different optimization methods of the two techniques. HyperArc relies on high modulation of MLC positions, dose rate, and gantry speed to achieve conformity, particularly for irregular or clustered lesions. This results in higher complexity, smaller apertures, increased leaf speed, and higher MUs. On the other hand, OptDCA, while using the same arc geometry, constrains modulation through the aperture shape controller and MU objectives, resulting in smoother MLC trajectories and larger effective apertures. Notably, compared with forward-planned DCA, OptDCA improves dosimetric performance through limited optimization, but it does not achieve the same level of flexibility as fully optimized VMAT.

The OptDCA approach could be particularly suitable for SRS of multiple small and spatially dispersed brain metastases. In such cases, OptDCA maintains clinically acceptable conformity and dose gradients while significantly reducing plan complexity, MUs and delivery time. These characteristics improve delivery robustness and result in higher gamma passing rates, indicating enhanced treatment reliability. OptDCA may therefore be advantageous in clinical workflows where efficiency and deliverability are prioritized, such as treatments involving numerous targets or patients with limited tolerance for prolonged beam-on times. However, for large, irregular, or closely clustered lesions requiring highly modulated dose shaping and maximal normal tissue sparing, VMAT-based approaches such as HyperArc may remain preferable. Accordingly, OptDCA should be considered a complementary technique rather than a universal replacement for VMAT.

There are limitations to our study that should be acknowledged. Firstly, it was conducted with an analysis of a relatively small patient cohort (30 patients, 2–10 metastases), which may reduce its applicability. Additionally, total target volume is an important factor in multitarget SRS planning, as increasing cumulative volume is associated with greater low-dose exposure to normal brain tissue and higher modulation demands. In the present study, total target volumes were recorded and reported (Table A1); however, the analysis was not stratified by volumetric burden, as the primary objective was a direct technique-level comparison using identical targets for each patient. Accordingly, the findings should be interpreted in the context of volumetric heterogeneity, while volume-based stratification may be explored in future studies. Another limitation is that clinical outcome data were not included in this study, as the primary objective was a technical and dosimetric comparison of planning and delivery characteristics between HyperArc and OptDCA. The retrospective nature of the analysis, combined with the relatively small cohort size, precluded meaningful evaluation of clinical endpoints such as local control or radiation necrosis. Finally, the use of PCI and GI as measures of conformity has been known to be less precise in small targets of <2 cc, which may lead to uncertainty in comparison.

Future studies including larger and more heterogeneous groups of patients—including those with complexly shaped or clustered lesions—should validate these findings. In addition, studies of comparative long-term results, directly between OptDCA and VMAT for toxicity and tumour control, are needed to provide evidence-based policy for practice. Another open area is the design of hybrid approaches of OptDCA and VMAT techniques in the same treatment plan, depending on the complexity brought about by the lesions’ geometry. These measures would optimize plan quality best and minimize delivery uncertainty, eventually leading to more efficient and safer radiosurgery treatment.

## 5. Conclusions

This study demonstrates that OptDCA planning can achieve a clinically acceptable plan quality for single-isocenter SRS of multiple brain metastases while substantially reducing plan complexity compared with VMAT-based approaches. OptDCA provided comparable target coverage, conformity, and dose gradient metrics, alongside significantly lower MUs and improved delivery accuracy, indicating enhanced treatment efficiency and robustness. While VMAT remains advantageous for targets requiring extensive modulation, such as highly irregular targets, OptDCA represents a simpler and reliable alternative, offering a balanced trade-off between plan quality and deliverability. These findings support the argument for the wider clinical adoption of the technique while providing a basis for future research to further explore the use of the technique in combination with VMAT or precisely identify clinical scenarios where one technique is preferable over the other.

## Figures and Tables

**Table 1:** Patient distribution by the number of metastases.

No. of Lesions	No. of Patients
2	11
3	10
4	3
5	3
6	1
7	1
8	-
9	-
10	1

**Table 2:** For both techniques, the median values of PCI, GI, MUs, V12, and V20 are given, and the range of the values is provided in parentheses.

Parameters	HyperArc	OptDCA	*p*-Value
PCI	0.72 (0.41–0.9)	0.71 (0.15–0.91)	**0.011**
GI	4.09 (2.52–89.53)	3.88 (1.4–57.67)	0.181
Target Coverage (%)	98.09 (93.3–104.8)	98.17 (87–108.23)	0.357
MU_s_	5473 (1883.5–9497.7)	4735 (1408.8–9562.6)	**0.007**
V_12_(cc)	6.95 (3.18–17.4)	9.33 (3.64–25.26)	**<0.001**
V_20_(cc)	2.56 (0.85–6.01)	2.73 (0.88–8.1)	**0.001**

## Data Availability

Dataset available on request from the authors.

## Abbreviations

BM	Brain Metastases
SRS	Stereotactic Radiosurgery
LINAC	Linear Accelerators
MLC	Multileaf Collimator
VMAT	Volumetric Modulated Arc Therapy
DCA	Dynamic Conformal Arc
TPS	Treatment Patient System
FFF	Flattening Filter Free
PO	Photon Optimizer
MU	Monitor Unit
NTO	Normal Tissue Objective
PTV	Planning Target Volume
PCI	Paddick Conformity Index
GI	Gradient Index
GPR	Gamma Passing Rate
DVH	Dose Volume Histogram
PSQA	Patient Specific Quality Assurance
O4D	Octavius 4D
PI	Plan Irregularity
LS	Leaf Speed
AAV	Aperture Area Variability
LSV	Leaf Sequence Variability
MCS	Modulation Complexity Score
ALPO	Average Leaf Pair Opening
*SAS*	Small Aperture Score
*EM*	Edge Metrics
*EFS*	Equivalent Field Size

## Appendix A

**Table A1:** Overview of the main lesions characteristics in the study population.

No. of Patients	No. of Lesions	Volume of Each Lesion (cc)
1	3	2.73, 3.18, 0.76
2	5	0.1, 0.27, 0.36, 0.25, 0.05
3	2	0.26, 0.17
4	2	0.04, 0.02
5	10	0.6, 0.5, 6.4, 0.5, 0.4, 8.8, 0.4, 0.3, 0.7, 1
6	6	5.39, 0.05, 0.07, 2.37, 1.81, 5.28
7	3	3.41, 1.02, 3.24
8	4	0.03, 0.08, 0.06, 0.39
9	2	0.95, 0.1
10	3	5.15, 6.45, 0.1
11	2	1.45, 2.1
12	5	2.09, 4.73, 7.5, 1.05, 0.44
13	7	0.51, 0.48, 0.65, 0.33, 0.93, 0.26, 0.27
14	2	14.6, 8.5
15	5	1.51, 1.43, 0.09, 0.03, 0.05
16	2	4.6, 1.7
17	3	0.7, 2.18, 0.22
18	3	0.3, 1.2, 0.7
19	2	0.14, 0.12
20	2	4.8, 2.8
21	2	0.32, 0.62
22	3	8.08, 0.77, 0.51
23	4	2.37, 2.94, 4.48, 0.6
24	3	3.4, 2.5, 2.4
25	3	2.6, 14.1, 13.6
26	2	1.7, 0.3
27	2	0.13, 0.23
28	4	0.34, 0.06, 0.03, 0.04
29	3	4.06, 0.86, 0.55
30	3	2.85, 0.02, 0.02

**Table A2:** Plan complexity metrics formulas calculated [35].

Category	Complexity Metrics	Formula
Deliverability	PI	$BI_{ij}=\frac{PI_{ij}^{2}}{4\pi A_{ij}}$
	LS	$LS=\frac{1}{NL_{ij}}\sum_{l=1}^{NL_{ij}}\frac{\Delta y_{ijl}}{dt_{i}j}$
	AAV	$AVV_{ij}=\frac{AAV_{ik}+AAV_{ik+1}}{2}$
	LSV	$LSV_{ij}=\frac{LSV_{ik}+LSV_{ik+1}}{2}$
	MCS	$MCS_{ij}=LSV_{ik}\cdot AAV_{ij}$
	ALPO	${ALPO}_{MU}=\sum_{k=1}^{k=K}\frac{\sum_{i=1}^{i=I-1}\left[\sum_{j=1}^{j=J}{\left(\left\lceil X_{R}-X_{L}\right\rceil \right)}_{ij}\right]\cdot {MU}_{i,i+1}}{\left(\sum_{i=1}^{i=I-1}{MU}_{i,i+1}\right)\cdot \sum_{j=1}^{j=J}j}$
Accuracy	SAS	$SAS=\frac{1}{\sum ijNL_{ij}/2}\sum_{i=1}^{N_{A}}\sum_{j=1}^{NCA_{i}}\sum_{l=1}^{NL_{ij}}\left[LG_{ij}<n\right]$
	EM	$EM_{ij}=\frac{C_{1}Px_{ij}+C_{2}Py_{ij}}{BA_{ij}}$
	EFS	$EFS_{ij}=\frac{2L_{ij}W_{ij}}{L_{ij}+W_{ij}}$
